# A Comparison of Conical and Cylindrical Implants Inserted in an In Vitro Post-Extraction Model Using Low-Density Polyurethane Foam Blocks

**DOI:** 10.3390/ma16145064

**Published:** 2023-07-18

**Authors:** Luca Comuzzi, Margherita Tumedei, Natalia Di Pietro, Tea Romasco, Hamid Heydari Sheikh Hossein, Lorenzo Montesani, Francesco Inchingolo, Adriano Piattelli, Ugo Covani

**Affiliations:** 1 Independent Researcher, San Vendemiano-Conegliano, 31020 Treviso, Italy; luca.comuzzi@gmail.com; 2 Department of Medical, Surgical, and Dental Sciences, University of Milan, 20122 Milan, Italy; margherita.tumedei@unimi.it; 3 Maxillo-Facial Surgery and Dental Unit, Fondazione IRCCS Ca’ Granda Ospedale Maggiore Policlinico, 20122 Milan, Italy; 4 Department of Medical, Oral and Biotechnological Sciences, “G. D’Annunzio” University of Chieti-Pescara, 66013 Chieti, Italy; tea.romasco@unich.it (T.R.); hamidheydari93@gmail.com (H.H.S.H.); 5 Center for Advanced Studies and Technology-CAST, “G. D’Annunzio” University of Chieti-Pescara, 66013 Chieti, Italy; 6 Villa Serena Foundation for Research, Via Leonardo Petruzzi 42, 65013 Città Sant’Angelo, Italy; 7 Independent Researcher, 00187 Rome, Italy; lomonte@bu.edu; 8 Interdisciplinary Department of Medicine, University of Bari “Aldo Moro”, 70121 Bari, Italy; francesco.inchingolo@uniba.it; 9 School of Dentistry, Saint Camillus International University of Health and Medical Sciences, 00131 Rome, Italy; apiattelli51@gmail.com; 10 Facultad de Medicina, UCAM Universidad Católica San Antonio de Murcia, 30107 Murcia, Spain; 11 Department of Stomatology, Tuscan Stomatologic Institute, Foundation for Dental Clinic, Research and Continuing Education, 55041 Camaiore, Italy; covani@covani.it

**Keywords:** artificial bone, conical implants, cylindrical implants, dental implants, implant stability quotient, insertion torque, polyurethane, post-extraction sites, removal torque

## Abstract

Combining tooth extraction and implant placement reduces the number of surgical procedures that a patient must undergo. Thus, the present study aimed to compare the stability of two types of conical implants (TAC and INTRALOCK) and another cylindrical one (CYROTH), inserted with a range of angulation of 15–20 degrees in low-density polyurethane blocks (10 and 20 pounds per cubic foot, PCF) with or without a cortical lamina (30 PCF), which potentially mimicked the post-extraction in vivo condition. For this purpose, a total of 120 polyurethane sites were prepared (10 for each implant and condition) and the Insertion Torque (IT), Removal Torque (RT), and Resonance Frequency Analysis (RFA) were measured, following a Three-Way analysis of variance followed by Tukey’s post hoc test for the statistical analysis of data. The IT and RT values registered for all implant types were directly proportional to the polyurethane density. The highest IT was registered by INTRALOCK implants in the highest-density block (32.44 ± 3.28 Ncm). In contrast, the highest RFA, a well-known index of Implant Stability Quotient (ISQ), was shown by TAC implants in all clinical situations (up to 63 ISQ in the 20 PCF block without the cortical sheet), especially in lower-density blocks. Although more pre-clinical and clinical studies are required, these results show a better primary stability of TAC conical implants in all tested densities of this post-extraction model, with a higher ISQ, despite their IT.

## 1. Introduction

Nowadays, advances in clinical techniques and biomaterials have facilitated the broadening of indications for immediate implant treatment [1,2]. Over the years, different types of implants and their positioning and loading protocols have evolved from the first protocols with the aim to obtain faster and easier surgical treatment times [3]. The immediate placement of dental implants in extraction sockets was described for the first time by Schulte and Heimke more than 40 years ago [4]. Since then, and as recently reported, preclinical, clinical, and radiological studies have allowed significant advances in understanding hard and soft tissue alterations in post-extraction sites [5]. Furthermore, it has been reported that the immediate dental implant loading procedure provides substantial advantages for the patient [6]. Thus, the immediate implant placement and provisionalization in post-extractive sockets have been proposed. As an example, Mura and collaborators [6], in a retrospective 5−year analysis of immediately loaded tapered implants placed in post-extraction sockets, have shown promising results concerning implant survival, soft tissue response, and peri-implant marginal bone conditions. Moreover, Han et al. [7] performed a comparison between survival, stability, and possible complications of immediately loaded tapered implants placed either in post-extraction or in healed sockets, and complications and failures were not reported to be significantly different between these two groups.

On the other hand, Mello et al. [8] conducted a systematic review with meta-analysis on the implant survival and possible peri-implant tissue modifications. Comparing immediate implant insertion in fresh extraction sockets and implant positioning in healed sites, they found that delayed implants reported a significantly higher survival in respect to immediate implants. In contrast, no differences were reported between the two groups as regards the marginal bone loss, the Implant Stability Quotient (ISQ) values, and the pocket probing depth. Similar results were reported in a recent systematic review [9] that compared post-extraction alveolar ridge preservation and the immediate implant insertion. Other authors, instead, asserted that an immediate implant insertion placement could be considered in post-extraction sites, since a limited amount of bone resorption was described [10]. However, other techniques, such as socket preservation using biomaterials and/or membranes, may be preferred when these conditions are not present [11].

Nowadays, polyurethane foam sheets have been used as a valuable substitute material for human bone in order to perform mechanical testing on instruments and orthopedic devices, as reported by the American Society for Testing and Materials [12], which recognized this material as a standard for in vitro tests. Recently, several authors started to use this artificial bone for mechanical testing on oral instruments and dental implants, especially for assessing implant primary stability [13,14]. Indeed, given the difficulties of working with human cadaver bones and animal bones, synthetic polyurethane foam has been widely used as alternative material in several biomechanical tests, as this material exhibits a similar cellular structure and consistent biomechanical characteristics [12,15,16,17,18]. In particular, a summary of the mechanical properties concerning the polyurethane foams used in this study (density, compression, and shear) and the corresponding ASTM F-1839-08 specifications have been reported in a previous study of this group [19]. Low- to high-density polyurethane foams are representative of different natural bone densities, according to the D1−D4 bone tissue classification proposed by Misch [20], since the ease and non-invasive nature of this model make it particularly valuable for predicting and evaluating the primary stability and osseointegration of implants in respect to other models, such as ex vivo or in vivo ones [21,22,23].

Specifically, in 2015, Kashi et al. [24] led an in vitro study with the aim to evaluate the primary stability of titanium implants inserted with different angle degrees in polyurethane foam sheets. For this purpose, polyurethane foam sheets mimicking artificial bone types II and IV, as well as angulations of 0, 10, and 20 degrees were used in this study, finding that implants placed with 10 degrees of angulation in a type II artificial bone showed a better primary stability. It should also be considered that, when using an in vitro polyurethane model also mimicking an extraction site, the implant design could have a pivotal role in achieving an adequate primary stability in challenging situations [25]. Moreover, Yim et al. [26], in a bovine bone in vitro study, reported that in peri-implant bone defects varying from 2 to 8 mm, decreased ISQ values and increased Periotest values were observed with the increase in the defect width.

Thus, the aim of the present study was to compare the stability of two types of conical implants (TAC and INTRALOCK) and a cylindrical one (CYROTH) when inserted with an angulation of 15–20 degrees in 10 and 20 Pounds per Cubic Foot (PCF) low-density polyurethane blocks with or without the presence of a cortical lamina (30 PCF in density), potentially mimicking the in vivo post-extraction condition. From this, the null hypothesis of the study would be the absence of differences in terms of Insertion Torque (IT), Removal Torque (RT), and Reference Frequency Analysis (RFA) values among conical and cylindrical implant macro-morphologies in order to guarantee a better implant behavior and primary stability in simulated extraction sites on polyurethane bone blocks.

## 2. Materials and Methods

### 2.1. Implant Description

Three types of implants were used for testing each experimental condition:

- TAC conical implants (Aon Implants, Grisignano di Zocco, Italy);
- INTRALOCK conical implants (Intra-Lock System Europa Spa, Salerno, Italy);
- CYROTH cylindrical implants (Aon Implants, Grisignano di Zocco, Italy).

All implants had the same dimensions (4 × 15 mm).

TAC implant macromorphology showed a more tapered and less aggressive collar shape, whereas threads were sharper and more aggressive. They presented a single-threaded design and there was a flat implant apex.

INTRALOCK implants had a more pronounced conical shape and the enlargement of the profile was 2 mm wider on the most coronal portion. The threads presented a triple pitch of the coil and there was a round apex.

**Figure 1:**
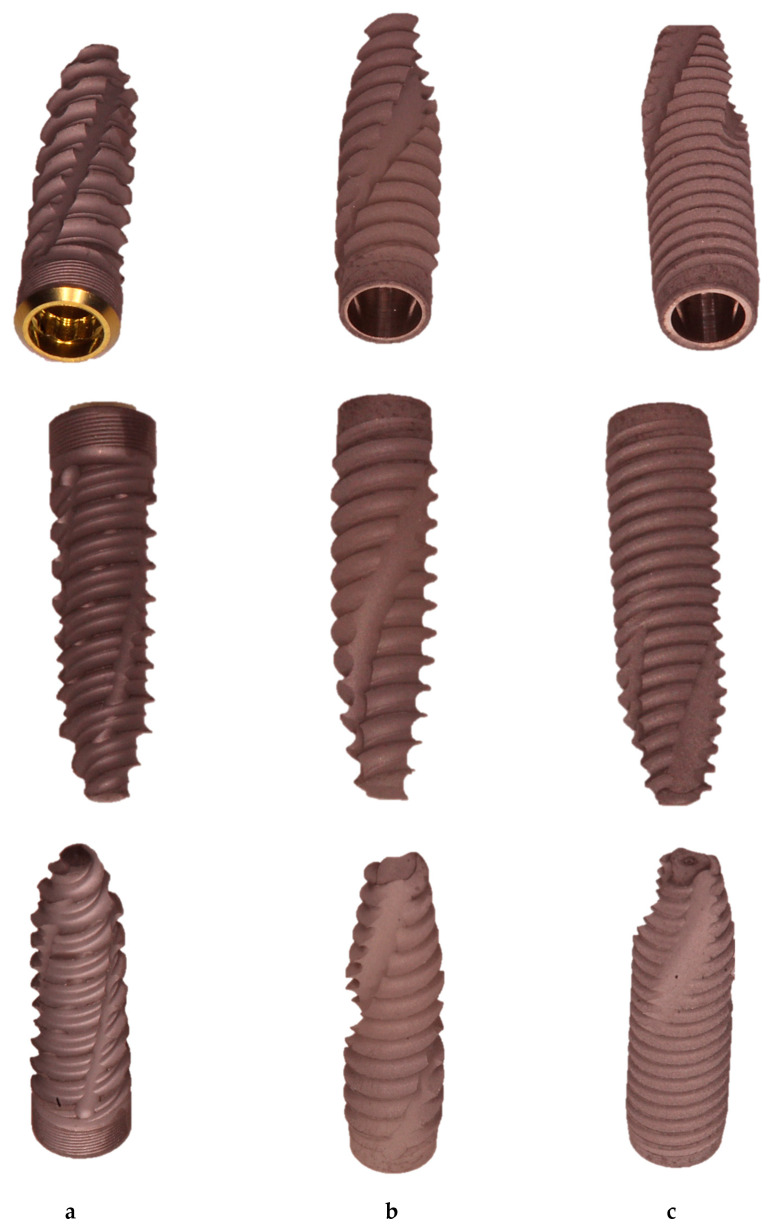
Representative images of the implants used in the present study: (**a**) INTRALOCK, (**b**) TAC, and (**c**) CYROTH implants from the bottom (first line), lateral (second line), and top (third line) views.

CYROTH cylindrical implants had a slightly tapered collar with less aggressive threads, which tended to compress and deform the material rather than cutting it. They also presented a conical apex (Figure 1).

### 2.2. Drilling Protocol and Implant Insertion

**Figure 2:**
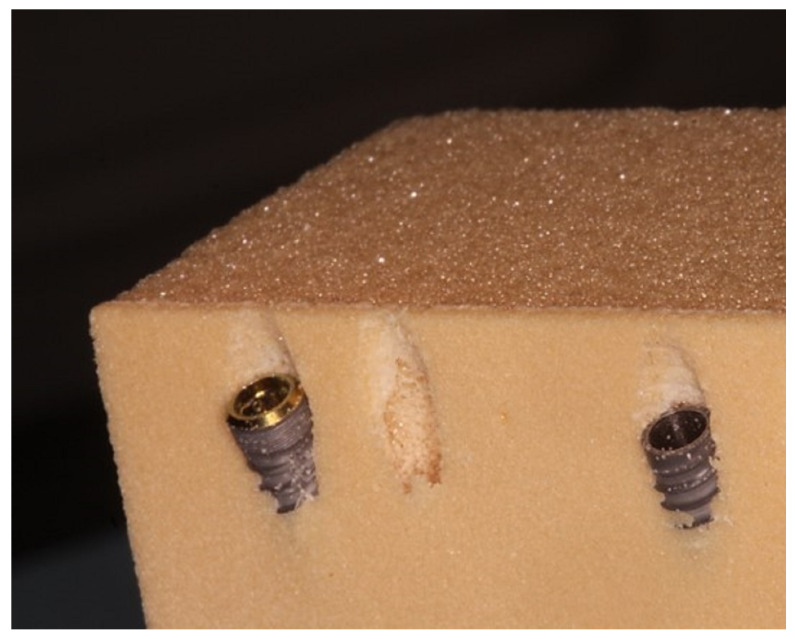
An example of the insertion of INTRALOCK (on the left) and TAC (on the right) implants up to 2 mm below the polyurethane profile and at 15–20 degrees of inclination.

**Figure 3:**
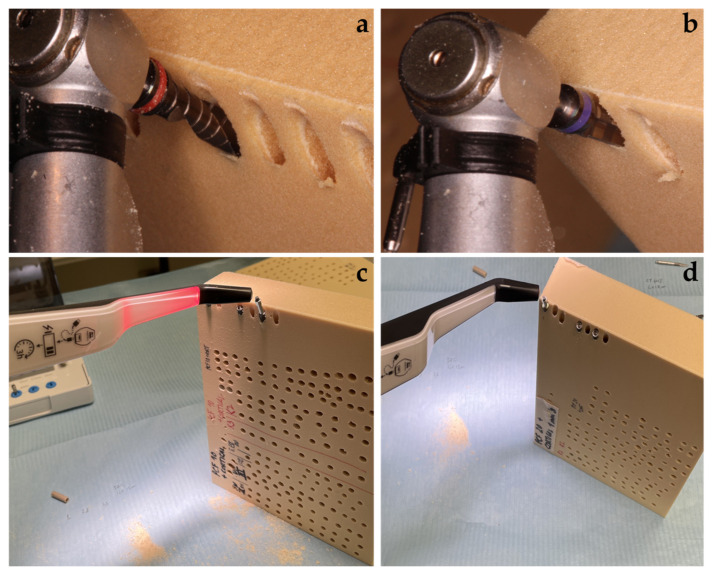
Representative images of the implant site preparation, implant insertion, and measurements: (**a**) Implant site preparation with 15–20 degrees of inclination; (**b**) Implant insertion; (**c**,**d**) Resonance Frequency Analysis (RFA) measurements.

The drilling protocol was performed by using an initial lanceolate bur at 300 rpm for all implants, followed by a 2.2 mm bur (AUN22300DR000, Aon Implants, Grisignano di Zocco, Italy) for TAC and CYROTH implants and a 2.0 mm bur (D-2015, Intra-Lock System Europa Spa, Salerno, Italy) for INTRALOCK implants, both used at 300 rpm. In order to finalize protocols, TAC and CYROTH implants were drilled with a 3.2 mm bur (AUN32000DR000, Aon Implants, Grisignano di Zocco, Italy), whereas INTRALOCK implants were drilled with a 4 mm conical bur (D-CT4D, Intra-Lock System Europa Spa, Salerno, Italy), both at 300 rpm. For this purpose, a Bien Air Chiropro (Bien Air SA, Bienne, Switzerland) surgical implant motor was used. The final implant insertion was performed at 30 rpm with a calibrated torque with a maximum range value of 50 Ncm and an inclination of 15–20 degrees; then, the Insertion Torque (IT) and the Removal Torque (RT) were evaluated in the last 1 mm during the implant seating, considered at 2 mm below the polyurethane block superficial profile. The n° 78 Smart Peg (Osstell AB, Gothenburg, Sweden) was used to evaluate the Resonance Frequency Analysis (RFA) values in the Bucco–Lingual (RFA-BL) and Mesial–Distal (RFA-MD) orientations (Figure 2 and Figure 3).

The protocol described above was conducted in order to mimic an immediate post-tooth extraction condition with implant placement in an aesthetic zone. In particular, this aimed to represent the implant positioning in fresh sites, where the residual non-healed alveolar bone usually requires drilling through the palatal wall of the inclined socket.

As regards polyurethane foam blocks, they are constituted by a well-known material used to mimic the natural bone, since it has pronounced mechanical characteristics, avoiding human variables or particular handling and preservation treatments whilst preserving similar bone properties [12,13,27]. Nowadays, it is also preferred to cadaver or animal bones for ethical reasons, and it is used as an alternative material to perform biomechanical tests regarding orthopedic or dental medical devices [14,28].

**Figure 4:**
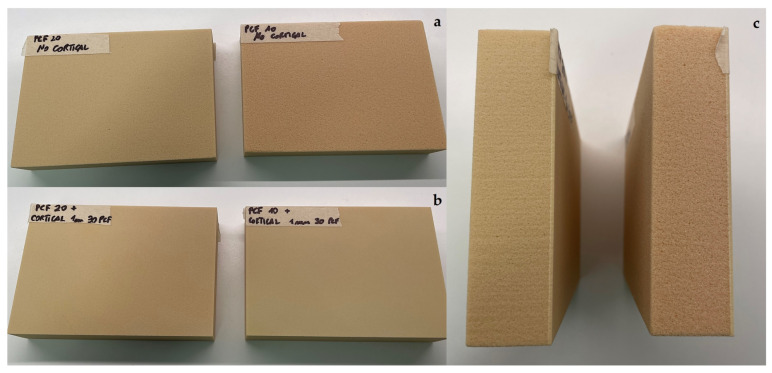
Representative images of the different blocks used: (**a**,**b**) polyurethane blocks of 20 and 10 Pounds per Cubic Foot (PCF) in density without and with the cortical sheets; (**c**) a detail of 20 and 10 PCF polyurethane blocks and the cortical sheets.

In this study 4-mm thick blocks with densities of 10 and 20 PCF (Sawbones Europe AB, Malmö, Sweden) were used, corresponding to a density of 0.16 g/cm^3^ and 0.32 g/cm^3^, mimicking D3 and D2 natural bone types, respectively. In addition, a 1-mm thick sheet with a density of 30 PCF (corresponding to a density of 0.48 g/cm^3^, similar to the D1 bone type) was added to the previous blocks when used to mimic the cortical bone [19] (Figure 4).

### 2.3. Study Design

To better clarify the dependent and independent variables analyzed in the present work, the different implant types, the different polyurethane densities, and the presence of a lamina could be identified as independent variables, whereas measurements of IT and RT have to be considered as dependent variables. In particular, the assessment of IT and RFA constitutes a non-destructive method to provide information on implant primary stability and survival [13,29], as the RT indirectly defines as well, representing a positive correlation with the degree of bone-to-impact contact (BIC) [23].

**Figure 5:**
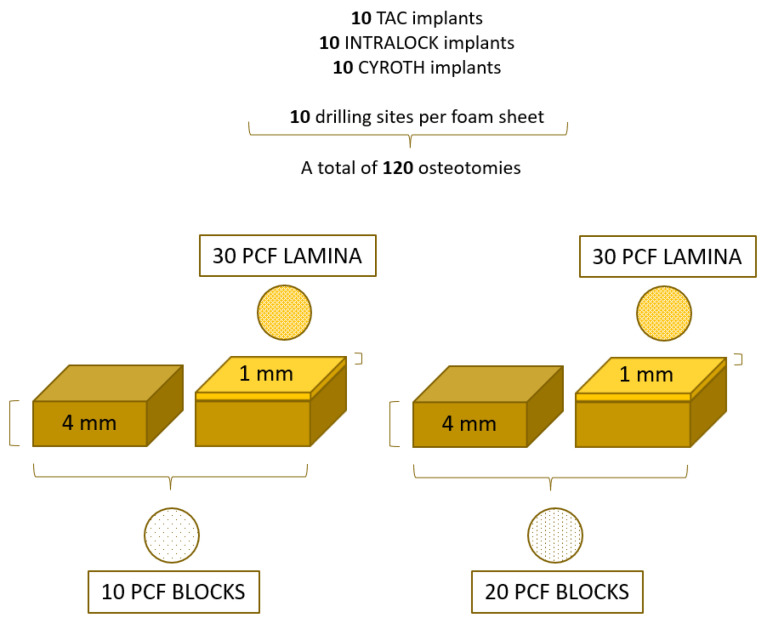
Schematic illustration of the osteotomies performed and the study design.

Thus, in Figure 5, the study design has been resumed: 10 implant sites were prepared for each implant type in all polyurethane densities, obtaining a total of 120 osteotomies.

### 2.4. Statistical Analysis

Power analysis and sample size planning were calculated using the ANOVA: fixed effects, special, main effects, and interactions statistical test. If we consider 4 conditions and 3 testing groups, the following chart turns out: effect size: 0.4, α err: 0.05; power (1−β): 0.9; numerator df: 6; number of groups: 12, using the G*Power 3.1.9.4 program to define it. The result of the minimum sample size necessary to achieve a statistically significant output was 116 implant sites and a total of 120 sites were performed in this study. The Shapiro–Wilk test was applied to evaluate the normal distribution of data. Subsequently, the differences among IT, RT, and RFA values expressed by the study groups were evaluated using a Three-Way analysis of variance (ANOVA) test, followed by Tukey’s post hoc test. A *p*-value < 0.05 was considered statistically significant. The research data and the statistical analysis were elaborated using the statistical software package GraphPad 9.0 (Prism, San Diego, CA, USA). Data were expressed as the mean ± Standard Deviation (SD).

## 3. Results

The experimental results related to the IT, RT, RFA-BL, and RFA-MD values evaluation and comparison are reported in Table 1. These values were obtained from independent measurements related to different implants inserted in each artificial bone condition.

### 3.1. Insertion Torque Evaluation

IT values appeared to be directly proportional to the polyurethane density, showing lower values in the lowest-density block, of 10 PCF in density, without the cortical sheet for all implant types, with a mean ± SD of 6.39 ± 0.41, 7.44 ± 0.34, and 6.73 ± 0.16 Ncm for TAC, INTRALOCK, and CYROTH implants, respectively. Specifically, TAC implants showed the lowest IT value (5.90 Ncm), but without statistically significant differences in respect to the other implants in the lowest-density condition. On the other hand, higher values were found in the block of 10 PCF density with the cortical sheet (with a mean ± SD of 15.16 ± 0.30 Ncm for TAC, 16.23 ± 0.36 Ncm for INTRALOCK, and 17.06 ± 0.30 Ncm for CYROTH implants) not reporting significant differences among groups, in the block of 20 PCF density without the cortical sheet (with a mean ± SD of 24.93 ± 0.34 Ncm for TAC, 23.83 ± 0.59 Ncm for INTRALOCK, and 26.42 ± 0.96 Ncm for CYROTH implants), reporting significant differences only between INTRALOCK and CYROTH (*p* < 0.0001), as well as in the block of 20 PCF density with the cortical sheet (with a mean ± SD of 24.63 ± 0.72 Ncm for TAC, 32.44 ± 3.28 Ncm for INTRALOCK, and 26.87 ± 1.04 Ncm for CYROTH implants). In particular, in this latter condition, INTRALOCK implants showed the highest IT value (37.20 Ncm), exhibiting significant differences in respect to the other implants (*p* < 0.0001).

**Figure 6:**
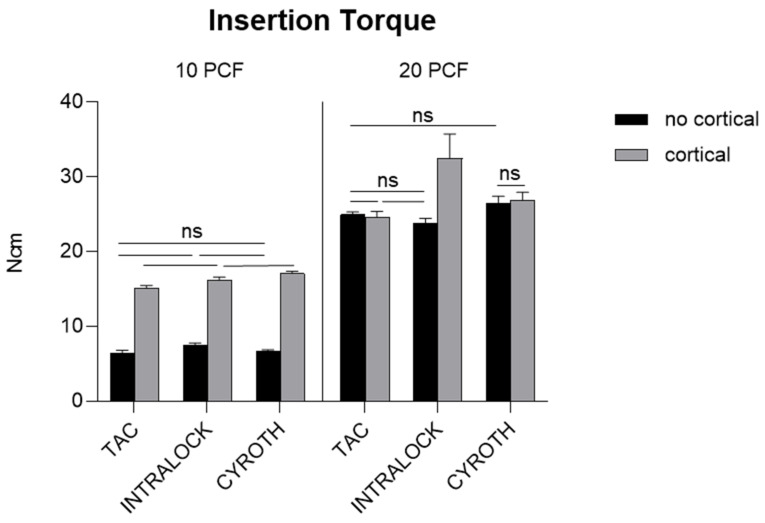
Bar graphs related to the distribution of Insertion Torque (IT) values expressed by each implant type in the different artificial bone conditions. Data were expressed as means ± Standard Deviation (SD). Non-significant differences were reported as “ns”, whereas the other comparisons were considered significant with a *p* < 0.05.

Figure 6 reports all the statistically significant and non-significant differences concerning IT measurements expressed by the implant types in the different experimental artificial bone densities.

Statistically significant higher values (*p* < 0.01) were found for CYROTH implants when inserted in the block of 10 PCF density with the cortical sheet and in the blocks of 20 PCF density without the cortical sheet (*p* < 0.0001) compared to TAC and INTRALOCK implants, respectively, as well as for those inserted in the block of 20 PCF density with the cortical sheet when compared to TAC implants (*p* < 0.001). As previously stated, INTRALOCK implants showed a statistical significance (*p* < 0.0001) only when inserted in the highest-density block, conversely reporting comparable results to the other implants in the blocks of 10 PCF density with and without the cortical sheet. Comparing blocks with the same density, added or not with the cortical sheet, statistical significances were found for all the implant types except for TAC and CYROTH in the block of 20 PCF in density. Similarly, each implant type inserted in the 20 PCF density blocks, with or without the cortical sheet, reported statistically significant higher values if compared with the corresponding one inserted in the 10 PCF blocks, added or not with the cortical sheet.

Overall, TAC implants resulted in slightly lower IT values in all the experimental conditions, except for the blocks of 10 and 20 PCF densities with the cortical sheet. However, they exhibited good IT values in all situations (14.7–25.5 Ncm) that were compatible with the mechanical implant stability, except for the block of 10 PCF density without the cortical sheet but showing no statistical differences with other implants’ values.

### 3.2. Removal Torque Evaluation

RT values were proportional to the polyurethane density as well, showing the highest values in the block of 20 PCF with the cortical sheet (with a mean ± SD of 21.60 ± 0.75 Ncm for TAC, 22.41 ± 0.72 Ncm for INTRALOCK, and 21.15 ± 1.14 Ncm for CYROTH implants) and the lowest ones in the block of 10 PCF density without the cortical sheet (with a mean ± SD of 4.81 ± 0.10 Ncm for TAC, 4.90 ± 0.00 Ncm for INTRALOCK, and 4.95 ± 0.08 Ncm for CYROTH implants). TAC implants showed the lowest RT values in the latest mentioned block (4.70 Ncm), whereas the highest results were reported by INTRALOCK implants in the thickest block of 20 PCF density with the cortical sheet (23.50 Ncm).

**Figure 7:**
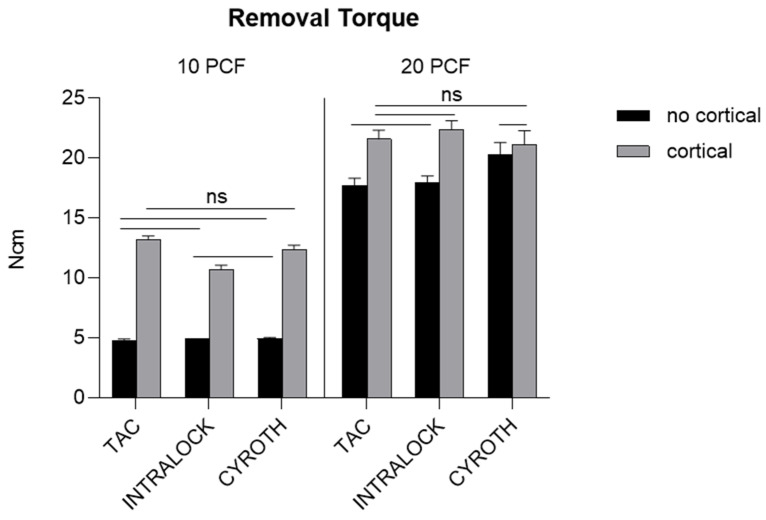
Bar graphs related to the distribution of Removal Torque (RT) values expressed by all the implant types in different artificial bone conditions. Data are expressed as means ± SD. Non-significant differences were reported as “ns”, whereas the other comparisons were considered significant with a *p* < 0.05.

Figure 7 shows that the RT values of all implant types inserted in the block of 10 PCF density without the cortical sheet were very low (about 5.00 Ncm), without reaching a statistical significance among groups.

INTRALOCK and TAC implants reported comparable values in all experimental conditions, except for the 10 PCF density block with the cortical sheet, where both TAC and CYROTH implants showed significantly higher results (*p* < 0.0001). CYROTH implants also showed significantly higher RT values in respect to both other implants in the block of 20 PCF density without the cortical sheet (*p* < 0.0001). On the other hand, INTRALOCK implants reported the highest results in the block of 20 PCF density with the cortical sheet but showing a statistical significance only when compared to CYROTH implants (*p* < 0.001). In addition, for each implant type, the RT values registered in the 20 PCF blocks with and without the cortical sheet were significantly higher than those registered by the same implant in the 10 PCF blocks, and if considering each implant inserted in the same-density blocks but with or without the cortical sheet, the RT values showed by the block with the cortical always reported significantly higher results, except for CYROTH implants in the 20 PCF blocks.

For all implants, the RT was always lower than the corresponding IT. Higher differences between IT and RT values were found for INTRALOCK implants (more than 10 Ncm in the 20 PCF density block with the cortical sheet) compared with TAC and CYROTH implants (4–6 Ncm lower). In the lowest-density block there were lower differences between IT and RT values.

### 3.3. Resonance Frequency Analysis Evaluation

**Figure 8:**
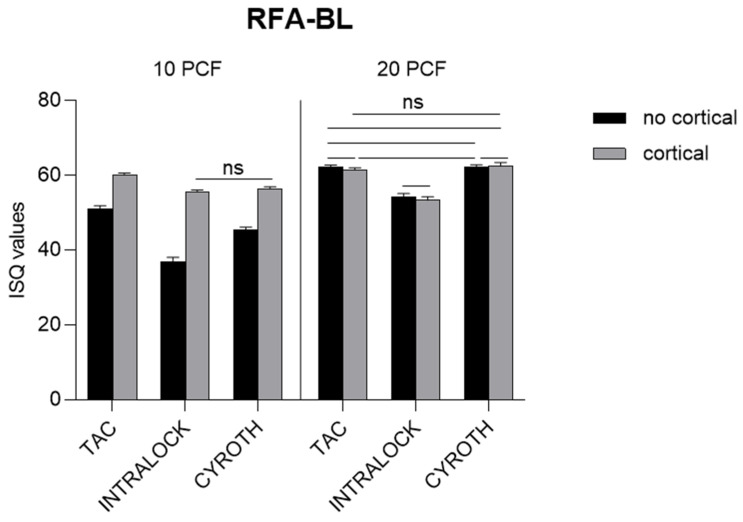

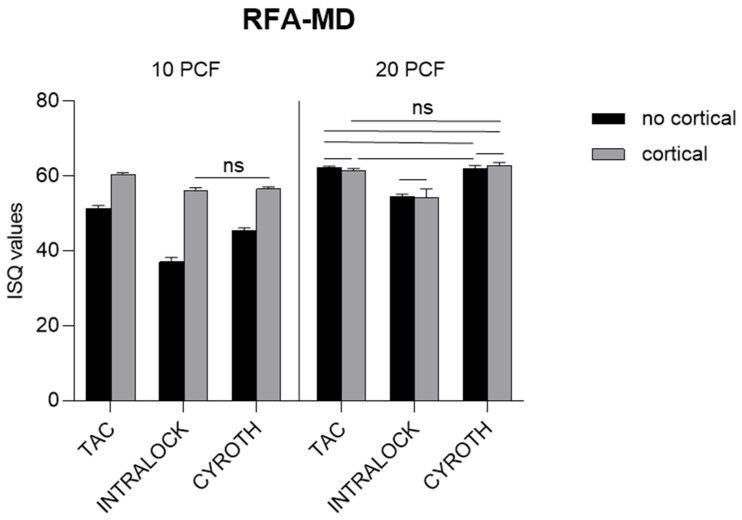
Bar graphs related to the distribution of RFA values in the Bucco–Lingual (BL, in the first line) and Mesial–Distal (MD, in the second line) orientations expressed by each implant type in the different artificial bone conditions. Data are expressed as means ± SD. Non-significant differences were reported as “ns”, whereas the other comparisons were considered significant with a *p* < 0.05.

RFA values, instead, were consistently higher for conical TAC implants in all the experimental conditions, especially in the lowest-density blocks (for example, with a mean ± SD of 51.20 ± 0.92 ISQ in the 10 PCF density block without the cortical sheet and 60.40 ± 0.52 ISQ in the 10 PCF density block with the cortical sheet, compared to 37.00 ± 1.25 and 56.00 ± 0.82 ISQ of INTRALOCK, and 45.30 ± 0.82 and 56.50 ± 0.53 ISQ of CYROTH implants in the same conditions), always reaching statistical significance (*p* < 0.0001). Only the 20 PCF density blocks, with and without the cortical sheet ISQ values, were similar for conical TAC (61.50 ± 0.53 and 62.20 ± 0.42 ISQ, respectively) and cylindrical CYROTH implants (62.90 ± 0.74 and 62.10 ± 0.74 ISQ, respectively); both were significantly higher than those of INTRALOCK implants (Figure 8).

Related to the 10 PCF block without the cortical sheet, INTRALOCK implants exhibited significantly lower ISQ values compared to CYROTH implants (*p* < 0.0001); this was different from the same implants in the 20 PCF block with the cortical sheet. In addition, TAC implants also reported statistically significant higher values in the latest mentioned condition.

Overall, only between the lowest-density blocks (10 PCF with and without the cortical sheet) did all implants show a statistical significance (*p* < 0.0001).

## 4. Discussion

In light of what the above results, the null hypothesis of the study (which considered the absence of differences in terms of IT, RT, and RFA values expressed by these conical and cylindrical implants in order to guarantee a better primary stability in the simulated extraction sites of the tested polyurethane foams) could be considered rejected.

In detail, primary stability is considered as the crucial factor to reach implant success and it was demonstrated to be mostly affected by implant macro-geometry and IT [30,31]. In particular, reaching an ideal primary stability in the posterior maxilla, corresponding to a D3 bone, represents a key factor for an immediate implant loading protocol, due to the low density of the bone [32]. Thus, in this in vitro study, the effects of different dental implant macro-morphologies on the IT, RT, and RFA, that directly or indirectly represented the implant primary stability, has been evaluated after their insertion in polyurethane foam blocks with different densities and simulating poor natural bone and post-extraction sites.

In the past, other authors [33] proposed the use of a tapered implant shape in order to improve the primary stability in a low-quality bone, since this macro-geometry was able to increase the pressure on the cortical bone in poor-bone regions. This fact could be relevant when implants are immediately or early loaded in poor-quality bone districts.

In this study, TAC implants are characterized by a more tapered shape and a less aggressive coronal portion compared to the other implants used. As a result, the implant insertion proceeded easily and progressively increased the IT until the final position, without undergoing deviations and preserving the surface profile of the material. Compared to INTRALOCK and CYROTH implants, there was a reduction in IT values that, however, allowed a more precise implant positioning without affecting the prepared site or excessively stressing the internal portion of the Cone–Morse connection. Interestingly, the registered ISQ values were the highest in all situations, even in low-density blocks (51.20 ± 0.92, 60.40 ± 0.52, 62.30 ± 0.48, and 61.50 ± 0.53 ISQ for 10 and 20 PCF density blocks without and with the cortical sheet, respectively), more likely due to the precise fitting of the implant during the insertion process, without being subjected to deviations. Regarding the implant threads and their difference from the INTRALOCK profile and apex, these implants had a more cutting and aggressive thread profile. This enabled them to penetrate the polyurethane material even without the use of a drilling protocol. This behavior could be especially useful in post-extraction conditions since it could help to direct the implant insertion and its adjustment when necessary, without the affection of the coronal portion.

On the other hand, the INTRALOCK macro-geometry is more conical than that of TAC implants and it has a profile that is 2 mm wider in the most coronal portion. This latter characteristic could be probably responsible for the higher IT values in the 20 PCF density block with the cortical sheet. In fact, the torque value increases when this part of the implant engages the polyurethane, even if it may cause a slight deviation of the implant during the insertion towards the extraction defect. This phenomenon is frequently seen when, inserting a post-extraction implant in the maxilla of a patient, the implant used has a wider profile in its coronal portion and meets a higher density in the palatal bone [34]. This fact could also contribute to decreasing the ISQ values, because the implant does not exactly fit into the prepared site, but undergoes a slight deviation, in part losing contact with the polyurethane. This phenomenon becomes more evident in lower-density blocks (36.80 ± 1.23 ISQ in the 10 PCF block without the cortical sheet).

CYROTH cylindrical implants, instead, presented a tapered coronal portion and a slight conicity when moving towards the apex, as well as less aggressive threads that tended to compress and deform the material rather than cutting it. This morphology resulted in good IT values, except for the lowest-density block (6.73 ± 0.16 Ncm), but produced a slight deviation toward the defect, as for INTRALOCK implants. This fact, together with the macro-geometry, could determine a significant decrease in ISQ values in respect to TAC implants, especially in 10 PCF density blocks (45.30 ± 0.82 and 56.50 ± 0.53 ISQ in blocks without and with the addition of the cortical sheet), but never lower than INTRALOCK implants, and they did not report statistical significance when inserted into the 10 PCF block with the cortical sheet.

As described in the literature [35], a high IT does not always correspond to a high ISQ, but high RFA values may be more desirable than a high IT for an immediate loading protocol to guarantee a better bone-to-implant contact. In this context, analyzing data reported in this study for post-extraction conditions, it was possible to assess that all implants presented IT values > 15 Ncm in all polyurethane densities, except for the 10 PCF block without the cortical sheet, with the highest values for INTRALOCK implants (32.44 ± 3.28 Ncm in the 20 PCF block with the cortical sheet) but considering a possible loss of direction during implant insertion. Contrarily, they always showed the lowest ISQ values in all situations (from 36.80 ± 1.23 to a maximum of 56.00 ± 0.82 ISQ in 10 PCF blocks without and with the cortical sheet, respectively) when compared to CYROTH and TAC implants. Specifically, these latter also showed higher ISQ values than CYROTH, especially when reaching significance in the lowest-density bones (51.20 ± 0.92 in comparison with 45.30 ± 0.82 ISQ and 60.40 ± 0.52 compared to 56.50 ± 0.53 in 10 PCF blocks without and with the addition of the cortical sheet, respectively). Thanks to their thread profile and apex shape, besides the conical macro-morphology, TAC implants may be considered as the best-performing implants for immediate loading simulation in post-extraction sites in all the artificial bone densities tested, as also corroborated by previous studies [36]. Probably, when using TAC implants in low-density bones, a higher value of under-preparation (from 3.2 mm to 2.2 mm) may also help to reach a higher IT value, which in combination with their high ISQ values makes these implants also appropriate for immediate loading in low-density bones [19].

In summary, it becomes necessary to shed light on the due limitations of this in vitro study that, albeit presenting comfortable data and a standardized artificial bone model [12], could obviously never be comparable to an ex vivo or clinical study. Even if the use of a polyurethane material could offer preliminary information on the biomechanical behavior of dental implants in different bone consistencies [14], further experimental and clinical studies are needed to corroborate these results on implant stability. The analyzed parameters could be affected by a patient’s physiological or pathological conditions and by other variables concerning bone density, such as the presence of natural bone or other grafting materials.

## 5. Conclusions

The present in vitro study performed in low-density polyurethane foam blocks demonstrated that the conical implant shape could be considered the best-performing in artificial post-extraction conditions, due to the higher primary stability values reported, despite the IT ones. In particular, the TAC implant’s macro-morphology reported the best results in terms of ISQ in all the polyurethane conditions and especially in lower-density blocks, aside from adequate values of IT and RT. Although further experimental studies are needed, in future a more standardized site under-preparation could help in obtaining higher IT values to make these implants ideal for an immediate loading protocol in low-density bones.

## Figures and Tables

**Table 1:** Statistic values of the Insertion Torque (IT), Removal Torque (RT), and Resonance Frequency Analysis in the Bucco–Lingual (RFA-BL) and Mesial–Distal (RFA-MD) orientations related to the different experimental conditions tested for each type of implant (TAC, INTRALOCK, and CYROTH). SD: Standard Deviation.

IT	10 PCF						20 PCF					
	No Cortical			Cortical			No Cortical			Cortical		
	TAC	INTRALOCK	CYROTH	TAC	INTRALOCK	CYROTH	TAC	INTRALOCK	CYROTH	TAC	INTRALOCK	CYROTH
Min	5.90	6.90	6.50	14.70	15.70	16.60	24.50	22.50	24.50	23.50	28.40	25.50
Max	6.90	7.80	6.90	15.70	16.70	17.60	25.50	24.50	27.40	25.50	37.20	28.40
Mean	6.39	7.44	6.73	15.16	16.23	17.06	24.93	23.83	26.42	24.63	32.44	26.87
SD (±)	0.41	0.34	0.16	0.30	0.36	0.30	0.34	0.59	0.96	0.72	3.28	1.04
RT	10 PCF						20 PCF					
	No Cortical			Cortical			No Cortical			Cortical		
	TAC	INTRALOCK	CYROTH	TAC	INTRALOCK	CYROTH	TAC	INTRALOCK	CYROTH	TAC	INTRALOCK	CYROTH
Min	4.70	4.90	4.80	12.70	10.00	12.00	16.60	16.90	18.60	20.50	21.50	19.70
Max	4.90	4.90	5.10	13.70	11.00	13.00	18.60	18.60	21.50	22.50	23.50	22.50
Mean	4.81	4.90	4.95	13.20	10.72	12.40	17.70	17.94	20.33	21.60	22.41	21.15
SD (±)	0.10	0.00	0.08	0.31	0.36	0.34	0.63	0.57	0.98	0.75	0.72	1.14
RFA—BL	10 PCF						20 PCF					
	No Cortical			Cortical			No Cortical			Cortical		
	TAC	INTRALOCK	CYROTH	TAC	INTRALOCK	CYROTH	TAC	INTRALOCK	CYROTH	TAC	INTRALOCK	CYROTH
Min	50.00	35.00	44.00	60.00	55.00	56.00	62.00	53.00	61.00	61.00	52.00	62.00
Max	52.00	38.00	46.00	61.00	56.00	57.00	63.00	55.00	63.00	62.00	55.00	64.00
Mean	51.00	36.80	45.40	60.20	55.50	56.40	62.30	54.20	62.20	61.50	53.30	62.60
SD (±)	0.82	1.23	0.70	0.42	0.53	0.52	0.48	0.92	0.63	0.53	0.95	0.84
RFA—MD	10 PCF						20 PCF					
	No Cortical			Cortical			No Cortical			Cortical		
	TAC	INTRALOCK	CYROTH	TAC	INTRALOCK	CYROTH	TAC	INTRALOCK	CYROTH	TAC	INTRALOCK	CYROTH
Min	50.00	35.00	44.00	60.00	55.00	56.00	62.00	53.00	61.00	61.00	52.00	62.00
Max	52.00	38.00	46.00	61.00	57.00	57.00	63.00	55.00	63.00	62.00	58.00	64.00
Mean	51.20	37.00	45.30	60.40	56.00	56.50	62.20	54.40	62.10	61.50	54.30	62.90
SD (±)	0.92	1.25	0.82	0.52	0.82	0.53	0.42	0.70	0.74	0.53	2.21	0.74

## Data Availability

Data are contained within the article and available on request from the corresponding author.
